# Biopsychosocial Late Effects After Cytoreductive Surgery and Hyperthermic Intraperitoneal Chemotherapy for Peritoneal Metastases from Colorectal and Appendiceal Cancer: A National Prospective Cohort Study

**DOI:** 10.1245/s10434-023-14618-6

**Published:** 2023-12-21

**Authors:** Rogini Balachandran, Henriette Vind Thaysen, Peter Christensen, Robert Zachariae, Lene Hjerrild Iversen

**Affiliations:** 1 Department of Surgery, Aarhus University Hospital, Aarhus, Denmark; 2 Department of Clinical Medicine, Aarhus University, Aarhus, Denmark; 3 Danish Cancer Society Centre for Research on Survivorship and Late Adverse Effects After Cancer in the Pelvic Organs, Aarhus University Hospital, Aarhus, Denmark; 4 Unit for Psychooncology and Health Psychology, Aarhus University Hospital, Aarhus, Denmark; 5 Department of Oncology, Aarhus University Hospital, Aarhus, Denmark; 6 Department of Psychology and Behavioural Sciences, Aarhus University, Aarhus, Denmark

**Keywords:** Late effect, Hyperthermic intraperitoneal chemotherapy, Cytoreductive surgical procedures, Colorectal cancer, Peritoneal metastases, Cancer survivors, Patient-reported outcome measures

## Abstract

**Background:**

Colorectal cancer with peritoneal metastases can be treated with cytoreductive surgery and hyperthermic intraperitoneal chemotherapy. Treatment may result in biopsychosocial late effects (LEs). We explored the frequency and severity of the following biopsychosocial LEs: anxiety, depression, fear of cancer recurrence (FCR), insomnia, fatigue, cognitive impairment, and pain, and evaluated their impact on quality of life (QoL).

**Method:**

This was a national prospective cohort study screening for LEs during the period January 2021–May 2023. Patients completed the following questionnaires: General Anxiety Disorder-7, Patient Health Questionnaire-9, FCR Inventory-Short Form, Insomnia Severity Index, Functional Assessment of Chronic Illness Therapy-Fatigue, cognitive impairment (six items from the European Organisation for Research and Treatment of Cancer Item Library), and the Rectal Cancer Pain Score. Preregistration was completed at ClinicalTrials.gov (NCT04956107).

**Result:**

In total, 99 patients were included. The mean age was 61 years and 57% were women. At 3 months after surgery, the frequent LEs were fatigue (72%), FCR (58%), and pain (48%), and at 12 months after surgery, the frequent LEs were FCR (65%), fatigue (40%), and insomnia (33%). More than half of the patients (54%) reported at least two LEs after 12 months. Patients with moderate-to-severe LEs reported a lower QoL than patients with no/mild LEs. Patients with no/mild LEs had a similar QoL as the Danish norm population.

**Conclusion:**

Biopsychosocial LEs were prevalent. The QoL of patients reporting LEs in the worst severity categories was negatively impacted. Screening and treatment for these LEs should be a focus in cancer survivor follow-up.

**Supplementary Information:**

The online version contains supplementary material available at 10.1245/s10434-023-14618-6.

Cytoreductive surgery (CRS) and hyperthermic intraperitoneal chemotherapy (HIPEC) is a curatively intended treatment option for selected patients with peritoneal metastases (PM) from colorectal cancer (CRC), which has significantly increased survival. A systematic review from 2020 reported a 5-year survival in the 19–51% range^1^ when CRC patients with PM were treated with CRS and HIPEC. CRS is in itself an extensive surgery with high postoperative morbidity, ranging from 23 to 66%,^1–5^ as well as a surgical treatment that enhances survival. Improvements in the oncological outcome will increase the number of long-term survivors who may be at risk of developing various late effects (LEs).

Relatively little is known about the development of the biopsychosocial LEs following CRS and HIPEC for advanced CRC with PM. For non-advanced CRC (stage I–III), we know that patients with poor sleep quality following their cancer treatment have an increased incidence of anxiety and depression.^6^ A recent prospective population-based study with 1535 patients assessed quality of life (QoL), anxiety, and depression following surgical or endoscopic treatment for pathological stage I–III CRC. The study revealed that up to 13% of the total cohort had persistent low QoL and high levels of psychological distress following their treatment.^7^ A review from 2021 found that up to 16% of CRC patients experienced high levels of ‘fear of cancer recurrence’ (FCR),^8^ and a large cross-sectional study from 2018 confirmed that patients with high levels of FCR experience a poorer QoL.^9^ Only a few studies have investigated psychological symptoms following CRS and HIPEC. These studies were predominately small, investigated very heterogeneous patient groups with different tumor origins, and investigated only a few LEs.^10,11^ Other LEs that may impact QoL include cognitive impairment and disturbed sleep. Cognitive impairments such as difficulties concentrating and remembering are commonly reported by cancer survivors, especially those returning to the labor market.^12^ Sleep disturbances such as insomnia, i.e., difficulties falling asleep and maintaining sleep during the night, are common and are associated with other LEs, including fatigue,^13^ another common LE after cancer treatment.

The aim of this study was to explore the frequency, severity, and change over time of biopsychosocial LEs (anxiety, depression, FCR, insomnia, fatigue, pain, and cognitive impairment) in patients having undergone CRS and HIPEC for CRC or appendiceal cancer. Furthermore, we aimed to explore the within- and between-patient variation of each LE investigated and to assess the impact of LE severity on QoL.

## Methods

### Study Design

This was a national, prospective, questionnaire-based cohort study.

### Study Population and Setting

The participating patients had undergone curatively intended CRS and HIPEC at Aarhus University Hospital (the only CRS and HIPEC center in Denmark) for (1) CRC with PM; (2) appendiceal cancer with PM; or (3) prophylactic CRS and HIPEC for perforated appendiceal cancer. PM was either synchronous or metachronous. PM diagnosed concurrently with the primary cancer or within an interval of up to 6 months is referred to as synchronous PM, while recurrence in the peritoneum diagnosed ≥6 months from the primary cancer is referred to as metachronous PM. The inclusion criteria were age ≥18 years and the ability to understand written Danish, whereas the exclusion criteria were patients undergoing CRS and HIPEC for cancers other than CRC and appendiceal cancer. Patients who had developed a recurrence after their surgery received questionnaires only until their recurrence was diagnosed. Patients who fell terminally ill during the follow-up were also excluded. The first patient underwent surgery on 12 November 2020 and had the first questionnaire distributed 3 months later in January 2021. The last included patient underwent surgery on 29 March 2023. The data collection period started on 25 January 2021 and ended on 16 May 2023. All patients were recruited before being discharged or transferred to another hospital.

### Timing of Questionnaires

Once recruited, patients were asked to complete questionnaires approximately 3, 6, and 12 months after their surgery. Patients who had a recurrence or failed to complete any questionnaire item were excluded from the study and did not receive the subsequent questionnaires. Patients with a recurrence were excluded since it was impossible to know if their symptoms at that time point were due to their recurrence or to LEs. Questionnaires were distributed online via the secure web application Research Electronic Data Capture (REDCap).^14^ If a questionnaire was not completed, two reminders were sent via REDCap at a 1-week interval. If the questionnaire had still not been completed, the primary investigator contacted the patient after the second week asking the patient to complete it.

### The Questionnaires

The questionnaire package included a number of validated patient-reported outcome measures (PROMs)^15–25^ as follows: (1) anxiety assessed using the 7-item Generalized Anxiety Disorder (GAD-7) questionnaire;^20^ (2) depressive symptoms assessed using the 9-item Patient Health Questionnaire (PHQ-9);^21^ (3) FCR assessed using the ‘Fear of Cancer Recurrence Inventory—Short Form’ (FCRI-SF);^22^ (4) insomnia assessed using the Insomnia Severity Index (ISI);^19^ (5) fatigue assessed using the Functional Assessment of Chronic Illness Therapy–Fatigue (FACIT-F);^15–17^ (6) pain assessed using the Rectal Cancer Pain Score;^18^ and (7) cognitive impairment assessed by six questions chosen from the European Organisation of Research and Treatment in Cancer (EORTC) Item Library. See Table 1 for an overview of the PROMs and their severity cut-off values. Based on their scores on anxiety, depression, insomnia, and fatigue, patients were categorized as having mild, moderate, or severe levels of these LEs. For FCR, patients were categorized as having no, mild, or severe FCR. Pain was categorized as no, minor, or major pain, and cognitive impairment was dichotomized into no cognitive impairment or cognitive impairment. QoL was investigated with the EORTC QLQ-C30 version 3.0, which includes five functional scales, three symptom scales, six single items, and global health status/QoL. The symptom scales and single items are mostly organ-specific and were not included in this article. A high score on a functional scale represents a high level of functioning, and a high global health status/QoL score represents a high QoL. A change of 10 points or more was considered significant.^25^

**Table 1:** Overview of patient-reported outcome measures

Outcomes	PROMs	Severity scoring range and cut-off value
Anxiety	GAD-7	Range: 0–21
		0–4: No anxiety
		5–9: Mild anxiety
		10–14: Moderate anxiety
		≥ 15: Severe anxiety
Depression	PHQ-9	Range: 0–27
		0–4: No depression
		5–9: Mild depression
		10–14: Moderate depression
		≥ 15: Severe depression
Fear of cancer recurrence	FCR—short form	Range: 0–36
		≥ 13: Possibility of clinical level FCR (mild FCR)
		≥ 22: Clinical severity of FCR in need of specialized intervention (severe FCR)
Insomnia	ISI	Range: 0–28
		0–7: No insomnia
		8–14: Mild–moderate insomnia
		15–21: Moderate–severe insomnia
		22–28: Severe insomnia
Fatigue	FACIT-F	Range: 0–52
		> 42 points: No fatigue
		34–42: Mild fatigue
		23–33: Moderate fatigue
		≤ 22: Severe fatigue
Pain	Rectal cancer pain score	Range: 0–45
		0–7: No pain or impact on QoL
		8–17: Minor pain impact on QoL
		≥ 18: Major pain impact on QoL.
Cognitive impairment	Six questions from the EORTC item library	Range: 0-100
		> 75: No cognitive impairment
		≤ 75: Cognitive impairment

*PROMs* patient-reported outcome measures, *GAD-7* 7-item Generalized Anxiety Disorder, *PHQ-9* 9-item Patient Health Questionnaire, *ISI* Insomnia Severity Index, *FACIT-F* Functional Assessment of Chronic Illness Therapy–Fatigue, *EORTC* European Organisation for Research and Treatment of Cancer, *FCR* fear of cancer recurrence, *QoL* quality of life

### Study Endpoints

The primary endpoints were the proportions of patients reporting each of the selected LEs at 3, 6, and 12 months after their surgery, while secondary endpoints included self-reported QoL in patients with no/mild and moderate/severe LEs. QoL data were also compared with previously published normative data from a random sample of 1832 Danes from the general population.^26^ Finally, we investigated the proportion of patients reporting several, i.e., clusters of, LEs and within- and between-patient variation of the scores. Clusters were investigated as the proportion of patients reporting two or more LEs at a specific assessment time point.

### Statistical Analysis

Normally distributed data are presented as means with range or 95% confidence interval (Cl), and non-normally distributed data are presented as medians with interquartile range (IQR). Each of the LEs investigated are presented as categorical variables according to degree of severity based on established cut-off levels. A sensitivity analysis was performed on all patients who had completed both the 3- and 12-month questionnaire. Patients excluded due to death or recurrence were not included in this sensitivity analysis. This analysis yielded the same results as the results from paired analysis on all patients (data not shown). Hence, all patients were included in the descriptive analyses, but *t* tests were only performed on paired observations. Data were analyzed using STATA statistical software, version 17.0 (StataCorp LLC, College Station, TX, USA).

### Ethical Approvals

The present study was registered with the Danish Data Protection Agency (case #: 1-16-02-714-20) and preregistered with ClinicalTrials.gov (NCT04956107). Approval by a scientific Ethics Committee is not required in Denmark for questionnaire-based studies. This was confirmed by the local scientific Ethics Committee (case #: 1-10-72-181-20). Consent for participation was obtained in accordance with the ethical standards of the Helsinki Declaration of 1975.

## Results

### Included Patients

**Fig. 1:**
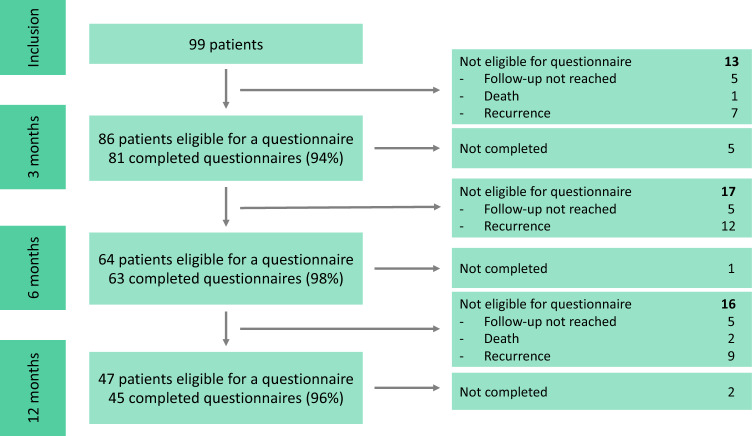
Number of patients

A total of 99 patients were included (see Fig. 1)*.* At each time assessment point, we did not distribute questionnaires to patients with a recurrence, patients who died, and patients who had not reached that assessment timepoint (follow-up not reached). Thus, questionnaires were distributed to the remaining eligible patients. At the 3-month timepoint, we distributed 86 questionnaires and received 81 completed questionnaires, yielding a response rate of 94% (81/86). For the 12-month timepoint, we distributed 47 questionnaires. The questionnaires were completed by 45 patients, yielding a 96% (45/47) response rate.

Characteristics of the included patients are shown in Table 2. The mean age (range) for patients who completed the questionnaire at 3 months after surgery was 60 years (35–79 years), and 46 (57%) were women. Most patients had a colon cancer 66 (81%) and 45 (56%) underwent surgery due to synchronous PM. In total, 25% of the patients developed a Clavien–Dindo complication of grade 3 or higher. In total, 94% of the patients received chemotherapy either prior to or after the CRS and HIPEC surgery. Patients who failed to complete the questionnaire generally appeared to be comparable with those who completed the questionnaire, with the exception that non-completers were slightly older and more likely to be men than completers were.

**Table 2:** Patient, tumor, and complication-related characteristics of the study cohort

	Patients completing the questionnaire at 3 months	Patients not completing the questionnaire at 3 months^a^
	[*n* = 81] (%)	[*n* = 18] (%)
*Sex*		
Female	46 (57)	8 (44)
Male	35 (43)	10 (56)
Age [mean (range)]	60 (35–79)	65 (45–78)
*Race*		
White	81 (100)	18 (100)
*Tumor origin*		
Colon cancer	66 (81)	13 (72)
Rectal cancer	6 (8)	3 (17)
Appendix cancer	9 (11)	2 (11)
*Timing of metastases*		
Synchronous	45 (56)	9 (69)
Metachronous	34 (42)	3 (23)
Prophylactic CRS and HIPEC	2 (2)	1 (8)
*Time between index surgery*^b^ *and CRS and HIPEC*		
Synchronous	75 days (range 5–260)	51 days (range 10–197)
Metachronous	530 days (range 123–2289)	730 days (range 261–2043)
Chemotherapy		
Both pre and post	17 (21)	4 (22)
Only pre	20 (25)	6 (33)
Only post	36 (44)	6 (33)
None (neither pre nor post)	5 (6)	1 (6)
Missing data	3 (4)	1 (6)
Complications – Clavien-Dindo grade 3 or higher		
Yes	20/81 (25)	5/13 (38)
No	61/81 (75)	8/13 (62)

Proportions are expressed as percentages

Mean values are expressed as ranges

^a^Patients not having completed the questionnaire at 3 months included patients who did not complete the questionnaire and patients who were excluded due to recurrence and hence did not receive the questionnaire

^b^Index surgery: surgery for the primary cancer

*CRS* cytoreductive surgery, *HIPEC* hyperthermic intraperitoneal chemotherapy, *Pre* Patients receiving chemotherapy prior to the CRS and HIPEC surgery, either as neoadjuvant treatment prior to the CRS and HIPEC surgery or as adjuvant treatment after their index surgery, *Post* Patients receiving adjuvant chemotherapy after the CRS and HIPEC surgery

### Frequency, Severity of Each Late Effect, and Change Over Time

**Fig. 2:**
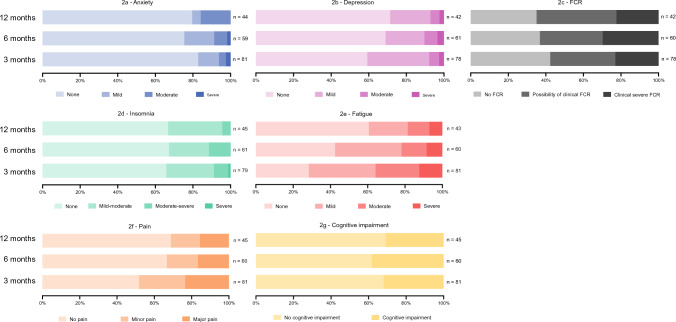
Frequency and severity of each late effect. *FCR* fear of cancer recurrence

The frequencies of each level of severity for each of the LEs at the three assessment time points are presented in Fig. 2. At 3 months after surgery, the three most frequently reported LEs were fatigue (72%), FCR (58%), and pain (48%); subsequently, at 12 months, the three most frequent LEs were FCR (65%), fatigue (40%), and insomnia (33%). For most LEs, frequencies decreased from 3 to 12 months (depression, insomnia, fatigue, pain); however, for two LEs, the frequency increased (anxiety, FCR), whereas frequency for cognitive impairment was stable from 3 to 12 months. Hence, the overall proportion of patients suffering from any degree of LEs decreased over time from 3 months after surgery to 12 months after surgery. The proportion of patients who reported no LEs more than doubled from 8.6% at 3 months after surgery to 20.0% at 12 months. Thus, 80% of the patients had at least one LE 1 year after surgery. Of these 80%, there was some variation in the proportions of patients scoring in the worse severity categories. Among patients who reported having anxiety 1 year after surgery, 16% had moderate–severe anxiety. Among patients with depression, 7% had a moderate–severe degree 1 year after surgery. For insomnia, the proportion of patients reporting either a moderate or severe degree was 4%, while the proportion of patients with moderate–severe fatigue 1 year after surgery was 19% and the proportion of patients with clinical severe FCR 1 year after surgery was 23%. For pain, 16% of patients reported major pain 1 year after surgery and 31% reported having cognitive impairment.

The mean score for each LE for each time assessment point is seen in Table 3. The improvements from 3 to 12 months reported for depression, insomnia, and fatigue were statistically significant.

**Table 3:** Mean values for each late effect at the three different time points

	3 months	6 months	12 months	Cut-off value	No. of patients included in the paired analyses	*p* value^a^
Anxiety	2.5 (1.6–3.3)	2.7 (1.7–3.7)	2.8 (1.5–4.0)	≥ 5	44	0.94
Depression	4.3 (3.4–5.2)	3.8 (2.7–4.9)	3.2 (2.0–4.4)	≥ 5	42	0.04
FCR	15.3 (13.5–17.1)	16.1 (13.9–18.3)	15.4 (13.1–17.7)	≥ 13	42	0.32
Insomnia	6.7 (5.6–7.9)	6.3 (4.8–7.7)	5.1 (3.8–6.5)	≥ 8	45	0.01
Fatigue	35.7 (33.6–37.8)	39.1 (36.4–41.7)	42.1 (39.4–44.8)	≤ 42	43	< 0.01
Pain	9.2 (6.9–11.6)	7.6 (4.6–10.5)	6.2 (3.2–9.3)	≥ 8	45	0.19
Cognitive impairment	81.6 (77.4–85.7)	76.0 (70.1–81.9)	77.8 (70.3–85.2)	≤ 75	45	0.44

Each score is expressed as mean value and 95% confidence interval

Cut-off values represent the predefined score for each LE, taken to indicate that the patient has the LE

^a^*p* values were calculated for paired observations comparing 3- and 12-month values. Thus, only patients completing both the 3- and 12-month questionnaires were included in these analyses

*FCR* fear of cancer recurrence, *LE* late effect

### Late Effect (LE) Clusters

The proportion of patients having at least two LEs changed from 78% at 3 months after surgery to 54% after 12 months. At 12 months after surgery, two patients (4%) had two LEs, 10 patients (22%) had three or four LEs, and 12 patients (28%) had five or more LEs. The following were the most frequent clusters 12 months after surgery: a combination of depression and cognitive impairment appeared among 42.9% of patients. The second and third most frequent combinations were FCR and insomnia (30.0%), and FCR and depression (28.9%). Furthermore, FCR and fatigue appeared in 28.2% of patients, and fatigue and cognitive impairment appeared in 27.9%. This made FCR and fatigue the most frequently co-occurring LEs, see Supplementary Fig. 1 for details on cluster distribution 3 and 12 months after surgery.

### The EORTC QLQ-C30-5 Functional Scales and Global Health Status/Quality of Life (QoL)

From 3 to 12 months after surgery, a clinically significant improvement, i.e., a score change of 10 points or more, was found for three functional scales—physical, role, and social functioning. For the remaining functional scales, we observed an improvement in global health status/QoL, a worsening in cognitive functioning, and similar values for emotional functioning from 3 to 12 months after surgery. However, none of these changes were clinically significant (see Table 4).

**Table 4:** The EORTC QLQ-C30-5 functional scales and global health status/QoL

	3 months	6 months	12 months	Danish population
Physical functioning	74.9 (70.8–79.0)	85.2 (81.0–89.5)	88.1 (83.8–92.4)	86 (20)
Role functioning	65.4 (58.6–72.2)	75.3 (67.9–82.6)	85.2 (79.1–91.3)	84 (27)
Emotional functioning	87.9 (84.2–91.6)	85.8 (80.9–90.7)	87.1 (81.4–92.9)	84 (19)
Cognitive functioning	81.5 (77.3–85.6)	76.1 (70.2–82.0)	77.0 (70.0–84.1)	87 (20)
Social functioning	80.2 (74.8–85.7)	83.3 (76.8–89.9)	90.3 (87.2–95.7)	90 (21)
Global health status	68.3 (64.1–72.5)	69.3 (63.7–74.8)	74.6 (68.9–80.3)	73 (23)

Each score is expressed as mean and 95% confidence interval

Normative data from a general Danish population are expressed as means and standard deviations

*QoL* quality of life

### QoL in Patients With/Without LEs

**Fig. 3:**
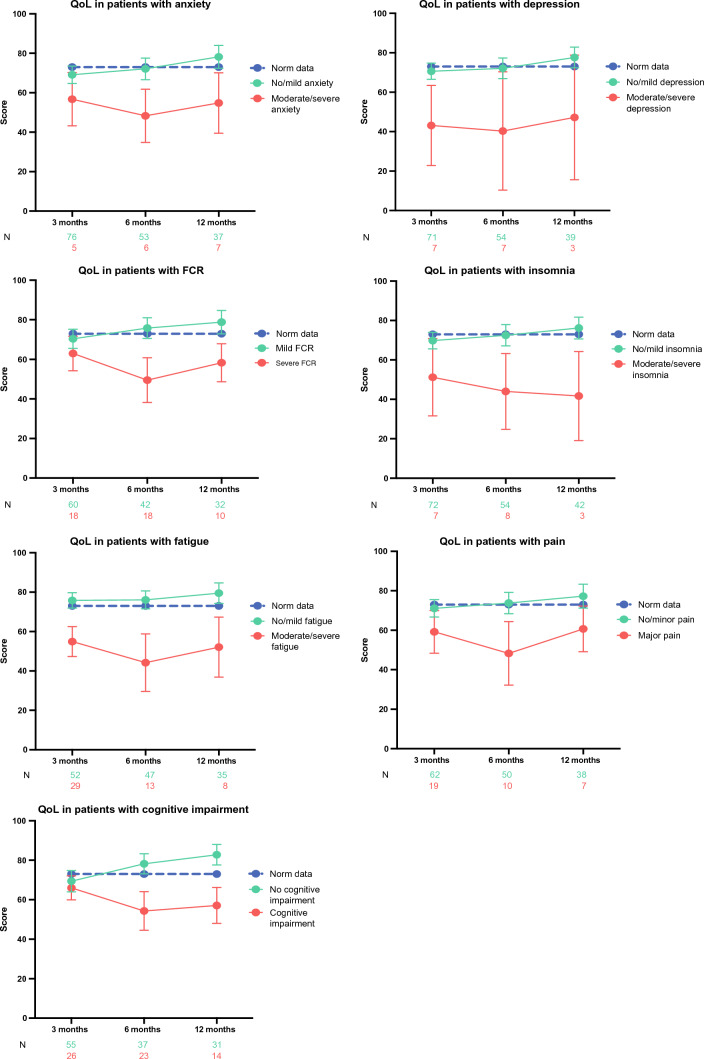
Each late effect versus quality of life. *QoL* quality of life, *FCR* fear of cancer recurrence. For anxiety, depression, insomnia and fatigue the red line represents patients scoring either moderate or severe and the green line represents patients scoring either as having no or a mild LE. For FCR, pain and cognitive impairment, patients scoring as having this issue according to the cutoff levels are presented with a red line and patients scoring as having no LE are presented with a green line. For all the figures the blue line represents norm data from a Danish population.

The QoL in patients with and without LEs, together with the normative data for the Danish norm population, are presented in Fig. 3. For all LEs, patients scoring in the worst severity categories for each LE had a lower QoL than patients with no/mild LEs. Patients with no/mild LEs had a QoL similar to that of the Danish norm population. Patients reporting cognitive impairment had a lower QoL than patients without cognitive impairment, who in turn seemed to report a better QoL than a normative sample from the Danish population.

### Score Variation

The individual scores of each patient for each LE are shown in Supplemental Fig. 1. For all LEs, each patient’s scores from 3, 6, and 12 months varied somewhat (within-patient variation). Patients scoring as having an LE at any given time point did not necessarily score as having this LE at another time point. Furthermore, the score at a specific time assessment point varied between patients (between-patient variation).

## Discussion

Even though the CRS and HIPEC procedure improves patient survival, the benefit of this procedure needs to be weighed against the risk of developing LEs and the QoL impact, as is the case for other types of complex surgery. The present study quantified the frequency of biopsychosocial LEs and explored associations with QoL after CRS and HIPEC for patients with PM from CRC or appendiceal cancer. LEs were reported in 80% of patients 12 months after surgery. Fatigue, FCR, and pain were the most prevalent LEs at 3 months after surgery, whereas FCR, fatigue, and insomnia were the most prevalent LEs at 12 months. On average, depression, insomnia, pain, and fatigue improved over time. In contrast, anxiety and FCR appeared to remain stable, whereas cognitive impairment worsened over time. The proportion of patients without LEs increased from 9% at 3 months after surgery to 20% at 12 months after surgery. Conversely, at 12 months after surgery, more than half of the patients (54%) reported at least two of the seven LEs, confirming that LEs were a persisting problem for many patients.

Our results also showed that LEs rarely occur in isolation but are often accompanied by other LEs. This highlights the importance of considering symptom clusters and exploring how individual symptoms may induce and perpetuate one another.^27^ Fatigue was one of the LEs that most frequently co-occurred with other LEs. One reason for this finding may be the multifactorial etiology of fatigue.^28,29^ Fatigue is reported in a moderate to severe level in 27% of CRC survivors^30^ and is one of the LEs that is most frequently reported among cancer survivors.^30,31^ In our study, approximately 40% of patients had some degree of fatigue 1 year after surgery. This number is higher than what the literature reports for non-advanced CRC survivors (27%), which could be due to the fact that patients in our study underwent more extensive surgery and had a more advanced cancer. Several LEs explored in this study have not previously been investigated in patients with CRS and HIPEC, hampering comparison with the existing literature. However, a recent individual participant data (IPD) meta-analysis of FCR found that up to 16% of non-advanced CRC patients experienced high levels of FCR.^8^ In our study, a larger proportion (22–25%) experienced severe FCR, a possible reason being that almost half (45%) of the patients in our sample underwent surgery for a metachronous/recurrent disease. In this patient group, higher levels of FCR may be expected. Several studies have investigated depression and anxiety, but they all focused on non-advanced CRC.^7,32–36^ One recent prospective study from 2021 including 825 colon cancer patients, and also a cross-sectional study from 2009, found that approximately 14–19% of patients experienced depression and anxiety 1 year after surgery.^7,36^ In two other studies reporting on depression in patients following a CRC diagnosis, 18% and 8% of patients reported moderate–severe depression^34^ 3–6 months and 6–12 months after diagnosis, respectively;^35^ however, these studies focused on patients with non-advanced colon cancer, which may possibly explain the higher proportion of patients with anxiety (21%) or depressive symptoms (29%) recorded in our sample 1 year after surgery. A 2022 study including non-advanced CRC survivors reported less fatigue but no changes in insomnia from 6 weeks to 24 months after surgery.^6^ We found improvements in both fatigue and insomnia after surgery. We only have data up to 12 months after surgery and are unaware if insomnia would have been stable if we had followed patients longer. A recent review from 2022 reported pain to be a frequent problem in patients who had undergone CRS and HIPEC; however, the paper also reported that pain decreased over time following surgery.^37^ The same pattern was observed in our sample. Previous research found that 49% of patients with lower gastrointestinal cancers (colon, rectum, and anal) reported cognitive impairment on average 2.4 years after treatment.^38^ This proportion is higher than the one recorded in our sample (29–37%) and this may be due to the longer observation time of the previous study. A national cross-sectional study on 9819 patients reported that patients with a cancer were 40% more likely to report having cognitive issues compared with patients with no cancer,^39^ and up to 35% of cancer patients have cognitive impairment months to years after their cancer treatment.^40^ This correlates well with our findings. We report that approximately 31% of patients experience cognitive impairment 1 year after surgery. Receiving chemotherapy is a known predictive factor for developing cognitive impairment.^40^ We were able to report on the proportion of patients receiving chemotherapy, but since patients received chemotherapy in different regions of Denmark, we did not have data regarding whether patients referred for chemotherapy actually completed all the planned sessions, or whether chemotherapy was prematurely terminated or administered in a low dosage. Since almost every patient (94%) had received chemotherapy, this could explain our findings with cognitive impairment not improving over time. Furthermore, we did not compare cognitive impairment between patients who did and did not receive systemic therapy as only five patients did not receive chemotherapy.

When evaluating the associations of the individual LEs with QoL, we observed a similar pattern for all seven LEs, indicating that patients who reported more severe LEs also had a poorer QoL than patients reporting no or mild LEs. Our results supported the findings from other studies with non-advanced colon cancer survivors, showing that patients with depression or anxiety after having undergone surgery also generally reported a poorer QoL.^9,41^ When comparing our results with normative data,^26^ we found that patients reporting no or mild LEs reported a QoL similar to that observed in a Danish norm population. Our results suggest that patients in our study, who underwent extensive surgery for advanced cancer, were able to maintain QoL levels similar to those observed in the general population, as long as they did not experience moderate–severe LEs. This further highlights the importance of screening for LEs and of offering relevant treatment to patients who develop LEs. While the result did not reach statistical significance, QoL in our study showed a trend towards improvement from 3 to 12 months. This correlates well with the findings of a recent review from 2020 reporting on QoL after CRS and HIPEC for a mixed cancer population, which included CRC patients. This review reports a noticeable decline in QoL within the first 3 months after surgery and then an improvement from 6 to 12 months after surgery.^2^

The main strengths of our study are its prospective design and broad range of LEs investigated. To our knowledge, the present study is the largest conducted on a range of LEs in patients who have undergone CRS and HIPEC for CRC or appendiceal cancer. We achieved a high response rate (93–96%), which may be as result of the prospective design and the fact that we asked patients to complete questionnaires shortly after surgery. The response rate was higher than the rates reported in the literature, ranging from 59 to 85%.^7,26,42,43^

Some limitations should also be noted. First, several patients were excluded due to recurrence/metastases (*n* = 28, 28%). We decided to exclude patients from the survey study if they had a recurrence; however, some early recurrences may not be detected by imaging and some recurrences identified by imaging may be asymptomatic. Furthermore, excluding detected recurrences from the study could therefore potentially introduce selection bias. On the other hand, as patients with recurrence would potentially have to undergo treatment and recurrence symptoms could falsely be registered as LEs, it seemed most appropriate to exclude patients with recurrence. However, if these excluded patients had developed more LEs than the non-recurrent patients, this would result in an underestimation of LE frequency.

Another limitation is that one PROM, the Rectal Cancer Pain Score, was validated for use in rectal cancer patients but most patients in our study were colon cancer survivors. Due to the lack of a PROM specifically designed for assessing pain in colon cancer survivors, and as some patients in our study had PM due to a rectal cancer, we decided to proceed with this PROM. Another potential limitation is the overlap between some items of the EORTC QLQ-C30 and the other PROMs used in our study, which may explain at least part of the association between LEs and QoL. Although there are several factors in the EORTC-QLQ-C30, such as social and role functioning, that do not align with the other LE PROMs used, we did observe associations between LEs and these functional domains.

A systematic review from 2010 on QoL among CRC survivors reported that younger age, physical problems, pain, low educational level, small social network, high body mass index, comorbidity, and having received a stoma were determining factors for having a poor QoL.^44^ This indicates that factors other than LEs may impact QoL; however, as multivariate analyses would require a considerably larger sample, we were unable to investigate other risk factors in the present sample.

Lastly, we did not collect preoperative data. We opted against this since patients who have received a recent diagnosis of CRC with PM could potentially respond to the diagnosis with high levels of anxiety, depression, fatigue, and insomnia. Furthermore, slightly less than half of the patients (42%) underwent surgery for metachronous PM. These patients would potentially respond with an elevated level of FCR because they had been diagnosed with a recurrence.

## Conclusion

Biopsychosocial LEs were frequent in CRC patients after surgery for PM. Whereas the proportion of patients experiencing LEs declined from 3 to 12 months after surgery, around three in every four patients (80%) continued to experience LEs at 12 months after surgery. Patients who developed LEs in the worst severity categories had a poorer QoL than those who did not, indicating a need for LE screening and treatment.

### Supplementary Information

Below is the link to the electronic supplementary material.

Supplementary file1 (DOCX 263 KB)

## References

[1] Mirnezami R, Mehta AM, Chandrakumaran K (2014). Cytoreductive surgery in combination with hyperthermic intraperitoneal chemotherapy improves survival in patients with colorectal peritoneal metastases compared with systemic chemotherapy alone. Br J Cancer.

[2] Leimkuhler M, Hentzen J, Hemmer PHJ (2020). Systematic review of factors affecting quality of life after cytoreductive surgery with hyperthermic intraperitoneal chemotherapy. Ann Surg Oncol.

[3] Chua TC, Yan TD, Saxena A, Morris DL (2009). Should the treatment of peritoneal carcinomatosis by cytoreductive surgery and hyperthermic intraperitoneal chemotherapy still be regarded as a highly morbid procedure?: A systematic review of morbidity and mortality. Ann Surg.

[4] Glehen O, Kwiatkowski F, Sugarbaker PH (2004). Cytoreductive surgery combined with perioperative intraperitoneal chemotherapy for the management of peritoneal carcinomatosis from colorectal cancer: a multi-institutional study. J Clin Oncol.

[5] van Leeuwen BL, Graf W, Pahlman L, Mahteme H (2008). Swedish experience with peritonectomy and HIPEC. HIPEC in peritoneal carcinomatosis. Ann Surg Oncol.

[6] Legg M, Meertens RM, van Roekel E (2022). The association between sleep quality and fatigue in colorectal cancer survivors up until two years after treatment: a cross-sectional and longitudinal analysis. Cancers.

[7] Qaderi SM, van der Heijden JAG, Verhoeven RHA, de Wilt JHW, Custers JAE, PLCRC study group (2021). Trajectories of health-related quality of life and psychological distress in patients with colorectal cancer: a population-based study. Eur J Cancer.

[8] Luigjes-Huizer YL, Tauber NM, Humphris G (2022). What is the prevalence of fear of cancer recurrence in cancer survivors and patients? A systematic review and individual participant data meta-analysis. Psychooncology.

[9] Mols F, Schoormans D, de Hingh I, Oerlemans S, Husson O (2018). Symptoms of anxiety and depression among colorectal cancer survivors from the population-based, longitudinal PROFILES Registry: prevalence, predictors, and impact on quality of life. Cancer.

[10] Loughney L, McCaffrey N, Timon CM (2020). Physical, psychological and nutritional outcomes in a cohort of Irish patients with metastatic peritoneal malignancy scheduled for cytoreductive surgery (CRS) and heated intrapertioneal chemotherapy (HIPEC): an exploratory pilot study. PLoS ONE.

[11] McQuellon RP, Loggie BW, Fleming RA, Russell GB, Lehman AB, Rambo TD (2001). Quality of life after intraperitoneal hyperthermic chemotherapy (IPHC) for peritoneal carcinomatosis. Eur J Surg Oncol.

[12] El-Shami K, Oeffinger KC, Erb NL (2015). American Cancer Society colorectal cancer survivorship care guidelines. CA Cancer J Clin.

[13] Coles T, Tan X, Bennett AV (2018). Sleep quality in individuals diagnosed with colorectal cancer: factors associated with sleep disturbance as patients transition off treatment. Psychooncology.

[14] Harris PA, Taylor R, Minor BL (2019). The REDCap consortium: building an international community of software platform partners. J Biomed Inform.

[15] Yellen SBCD, Webster K, Blendowski C, Kaplan E (1997). Measuring fatigue and other anemia-related symptoms with the Functional Assessment of Cancer Therapy (FACT) measurement system Author links open overlay panel. J Pain Symptom Manag.

[16] Smith E, Lai JS, Cella D (2010). Building a measure of fatigue: the functional assessment of Chronic Illness Therapy Fatigue Scale. PM R.

[17] Van Belle S, Paridaens R, Evers G (2005). Comparison of proposed diagnostic criteria with FACT-F and VAS for cancer-related fatigue: proposal for use as a screening tool. Support Care Cancer.

[18] Mortensen AR, Thyo A, Emmertsen KJ, Laurberg S (2019). Chronic pain after rectal cancer surgery—development and validation of a scoring system. Colorectal Dis.

[19] Bastien CHVA, Morin CM (2001). Validation of the Insomnia Severity Index as an outcome measure for insomnia research. Sleep Med.

[20] Spitzer RL, Kroenke K, Williams JBW, Löwe B (2006). A brief meassure GADS-7. Arch Intern Med.

[21] Kroenke KSR, Williams JBW (2001). The PHQ-9 validity of a brief depression severity measure-annotated. J Gen Intern Med.

[22] Simard S, Savard J (2015). Screening and comorbidity of clinical levels of fear of cancer recurrence. J Cancer Surviv.

[23] Hovdenak Jakobsen I, Jeppesen MM, Simard S, Thaysen HV, Laurberg S, Juul T (2018). Initial validation of the Danish version of the Fear of Cancer Recurrence Inventory (FCRI) in colorectal cancer patients. J Cancer Surviv.

[24] Fardell JE, Jones G, Smith AB (2018). Exploring the screening capacity of the Fear of Cancer Recurrence Inventory-Short Form for clinical levels of fear of cancer recurrence. Psychooncology.

[25] Osaba D, Rodregues G, Myles J, Zee B, Pater J (1998). Interpreting the significance of changes in health-related Quality-of-Life scores. J Clin Oncol.

[26] Juul T, Petersen MA, Holzner B, Laurberg S, Christensen P, Gronvold M (2014). Danish population-based reference data for the EORTC QLQ-C30: associations with gender, age and morbidity. Qual Life Res.

[27] Miaskowski C, Barsevick A, Berger A (2017). Advancing symptom science through symptom cluster research: expert panel proceedings and recommendations. J Natl Cancer Inst.

[28] Emery J, Butow P, Lai-Kwon J, Nekhlyudov L, Rynderman M, Jefford M (2022). Management of common clinical problems experienced by survivors of cancer. Lancet.

[29] Cella D, Lai JS, Chang CH, Peterman A, Slavin M (2002). Fatigue in cancer patients compared with fatigue in the general United States population. Cancer.

[30] Wang XSZF, Fisch MJ, O’Mara AM, Cella D, Mendoza TR (2014). Prevalence and characteristics of moderate to severe fatigue: a multicenter study in cancer patients and survivors. Cancer.

[31] Minton OSF, Radbruch L, Stone P (2012). Identification of factors associated with fatigue in advanced cancer: a subset analysis of the European Palliative Care Research collaborative computerized symptom assessment data set. J Pain Symptom Manag.

[32] Medeiros M, Oshima CT, Forones NM (2010). Depression and anxiety in colorectal cancer patients. J Gastrointest Cancer.

[33] Mosher CE, Winger JG, Given BA, Helft PR, O'Neil BH (2016). Mental health outcomes during colorectal cancer survivorship: a review of the literature. Psychooncology.

[34] Walling AM, Weeks JC, Kahn KL (2015). Symptom prevalence in lung and colorectal cancer patients. J Pain Symptom Manag.

[35] Lynch BM, Steginga SK, Hawkes AL, Pakenham KI, Dunn J (2008). Describing and predicting psychological distress after colorectal cancer. Cancer.

[36] Simon AE, Thompson MR, Flashman K, Wardle J (2009). Disease stage and psychosocial outcomes in colorectal cancer. Colorectal Dis.

[37] Balachandran R, Mogensen LZ, Christensen P, Thaysen HV, Iversen LH (2022). Organ-specific adverse effects after cytoreductive surgery with hyperthermic intraperitoneal chemotherapy. Ann Surg Oncol.

[38] Frick MA, Vachani CC, Hampshire MK (2017). Survivorship after lower gastrointestinal cancer: patient-reported outcomes and planning for care. Cancer.

[39] Jean-Pierre PWP, Ahles TA, Antoni M, Armstrong FD, Penedo F (2012). Prevalence of self-reported memory problems in adult cancer survivors—a national cross-sectional study. J Oncol Pract.

[40] Janelsins MC, Kohli S, Mohile SG, Usuki K, Ahles TA, Morrow GR (2011). An update on cancer- and chemotherapy-related cognitive dysfunction: current status. Semin Oncol.

[41] Aminisani N, Nikbakht H, Asghari Jafarabadi M, Shamshirgaran SM (2017). Depression, anxiety, and health related quality of life among colorectal cancer survivors. J Gastrointest Oncol..

[42] Juul T, Bräuner AB, Drewes AM (2021). Systematic screening for late sequelae after colorectal cancer-a feasibility study. Colorectal Dis.

[43] Custers JAE, Gielissen MFM, Janssen SHV, de Wilt JHW, Prins JB (2016). Fear of cancer recurrence in colorectal cancer survivors. Support Care Cancer.

[44] Jansen L, Koch L, Brenner H, Arndt V (2010). Quality of life among long-term (>/=5 years) colorectal cancer survivors—systematic review. Eur J Cancer.

