# Supplementary material for: Biopsychosocial Late Effects After Cytoreductive Surgery and Hyperthermic Intraperitoneal Chemotherapy for Peritoneal Metastases from Colorectal and Appendiceal Cancer: A National Prospective Cohort Study
Source: Ann Surg Oncol. 2023 Dec 21;31(3):1959–69. doi: 10.1245/s10434-023-14618-6 (PMC10838225; doi:10.1245/s10434-023-14618-6)
Supplement: Supplementary file 1 — Supplementary file1 (DOCX 263 KB) [file 10434_2023_14618_MOESM1_ESM.docx]

**Supplemental Figure 1 - Cluster distribution at 3 and 12 months after surgery**

**Supplemental Figure 2 - Within-patient variation and between-patient variation for scores**
